# Outbreak Caused by VIM-1- and VIM-4-Positive *Proteus mirabilis* in a Hospital in Zagreb

**DOI:** 10.3390/pathogens14080737

**Published:** 2025-07-26

**Authors:** Branka Bedenić, Gernot Zarfel, Josefa Luxner, Andrea Grisold, Marina Nađ, Maja Anušić, Vladimira Tičić, Verena Dobretzberger, Ivan Barišić, Jasmina Vraneš

**Affiliations:** 1 Biomedical Research Institute-BIMIS, University of Zagreb School of Medicine, 10000 Zagreb, Croatia; 2 Clinical Department for Clinical Microbiology, Infection Control and Prevention, School of Medicine, University Hospital Center Zagreb, University of Zagreb, 10000 Zagreb, Croatia; 3 Diagnostic and Research Institute for Hygiene, Microbiology and Environmental Medicine, Medical University Graz, 8010 Graz, Austria; gernot.zarfel@medunigraz.at (G.Z.); josefa.luxner@medunigraz.at (J.L.);; 4 University of Zagreb School of Medicine,10000 Zagreb, Croatia; 5 Department of Clinical Microbiology, Dr Andrija Štampar Teaching Institute of Public Health Zagreb, 10000 Zagreb, Croatia; maja.anusic@stampar.hr (M.A.); vladmira.ticic@stampar.hr (V.T.); jasmina.vranes@stampar.hr (J.V.); 6 Department of Molecular Diagnostics, Austrian Institute for Technology, 1210 Vienna, Austria; 7 Department of Medical Microbiology and Parasitology, University of Zagreb School of Medicine, 10000 Zagreb, Croatia

**Keywords:** *Proteus mirabilis*, multidrug resistance, VIM, CTX-M-15, epidemic spread

## Abstract

Background/objectives: *Proteus mirabilis* is a frequent causative agent of urinary and wound infections in both community and hospital settings. It develops resistance to expanded-spectrum cephalosporins (ESCs) due to the production of extended-spectrum β-lactamases (ESBLs) or plasmid-mediated AmpC β-lactamases (p-AmpCs). Recently, carbapenem-resistant isolates of *P. mirabilis* emerged due to the production of carbapenemases, mostly belonging to Ambler classes B and D. Here, we report an outbreak of infections due to carbapenem-resistant *P. mirabilis* that were observed in a psychiatric hospital in Zagreb, Croatia. The characteristics of ESBL and carbapenemase-producing *P. mirabilis* isolates, associated with an outbreak, were analyzed. Materials and methods: The antibiotic susceptibility testing was performed by the disk-diffusion and broth dilution methods. The double-disk synergy test (DDST) and inhibitor-based test with clavulanic and phenylboronic acid were applied to screen for ESBLs and p-AmpCs, respectively. Carbapenemases were screened by the modified Hodge test (MHT), while carbapenem hydrolysis was investigated by the carbapenem inactivation method (CIM) and EDTA-carbapenem-inactivation method (eCIM). The nature of the ESBLs, carbapenemases, and fluoroquinolone-resistance determinants was investigated by PCR. Plasmids were characterized by PCR-based replicon typing (PBRT). Selected isolates were subjected to molecular characterization of the resistome by an Inter-Array Genotyping Kit CarbaResisit and whole-genome sequencing (WGS). Results: In total, 20 isolates were collected and analyzed. All isolates exhibited resistance to amoxicillin alone and when combined with clavulanic acid, cefuroxime, cefotaxime, ceftriaxone, cefepime, imipenem, ceftazidime–avibactam, ceftolozane–tazobactam, gentamicin, amikacin, and ciprofloxacin. There was uniform susceptibility to ertapenem, meropenem, and cefiderocol. The DDST and combined disk test with clavulanic acid were positive, indicating the production of an ESBL. The MHT was negative in all except one isolate, while the CIM showed moderate sensitivity, but only with imipenem as the indicator disk. Furthermore, eCIM tested positive in all of the CIM-positive isolates, consistent with a metallo-β-lactamase (MBL). PCR and sequencing of the selected amplicons identified VIM-1 and VIM-4. The Inter-Array Genotyping Kit CarbaResist and WGS identified β-lactam resistance genes *bla*_VIM_, *bla*_CTX-M-15_, and *bla*_TEM_ genes; aminoglycoside resistance genes *aac(3)-IId*, *aph(6)-Id*, *aph(3″)-Ib*, *aadA1*, *armA*, and *aac(6′)-IIc*; as well as resistance genes for sulphonamides *sul1* and *sul2*, trimethoprim *dfr1*, chloramphenicol *cat*, and tetracycline *tet(J)*. Conclusions: This study revealed an epidemic spread of carbapenemase-producing *P. mirabilis* in two wards in a psychiatric hospital. Due to the extensively resistant phenotype (XDR), therapeutic options were limited. This is the first report of carbapenemase-producing *P. mirabilis* in Croatia.

## 1. Introduction

*Proteus mirabilis* is an opportunistic pathogen, belonging to the family *Enterobacterales*, and is widely distributed in the environment and the normal microbiota in the bowels of humans and animals [1]. Along with other *Enterbacterales*, it is considered to be an important causative agent of urinary tract infections (UTIs), particularly catheter-associated urinary tract infections (CAUTIs) due to its ability to form biofilms and infect wounds [2]. *P. mirabilis* is intrinsically resistant to tigecycline, polymyxins, tetracyclines, and nitrofurantoin [3] but does not have any intrinsic β-lactamase resistance.

Resistance to β-lactams in *P. mirabilis* is mediated by β-lactamases, enzymes that cleave β-lactam antibiotics, as well as the modification of penicillin-binding proteins (PBPs), upregulation of efflux pumps, and porin alteration or loss. According to the Ambler classification that is based on the molecular structure, β-lactamases are classified as classes A, B, C, and D. The β-lactamases belonging to classes A, C, and D contain serine in the active site, while class B contains zinc, which is involved in catalysis and is susceptible to metal chelators such as EDTA [4]. The β-lactamases encountered in *Proteus* species are broad-spectrum penicillinases (TEM-1, TEM-2, SHV-1, CARB, and OXA-1-4), extended-spectrum β-lactamases (ESBLs), inhibitor-resistant TEM β-lactamases (IRTs), plasmid-mediated AmpC β-lactamase (p-AmpC), and carbapenemases [3]. ESBLs hydrolyze penicillins, all cephalosporins, and monobactams, whereas p-AmpC inactivates expanded-spectrum cephalosporins or ESCs (ceftazidime, cefotaxime, ceftriaxone) and cephamycins but spares carbapenems and cefepime. Carbapenems are potent β-lactam antibiotics and the last-resort treatment for infections due to ESBL- and AmpC-producing organisms [5,6]. Inappropriate use of carbapenems leads to the proliferation of carbapenemases. Carbapenemases found in *P. mirabilis* (such as VIM, IMP, and NDM) belong predominantly to the Ambler class B metallo-β-lactamases (MBLs). Additionally, class D carbapenem-hydrolyzing oxacillinases (CHDLs), including OXA-48, OXA-181, OXA-23, and OXA-58, are encountered, while class A enzymes like KPC are only rarely detected [3,7]. Resistance to aminoglycosides in *P. mirabilis* is most frequently caused by enzymes that modify aminoglycosides and render them inactive. Adenyltransferases are encoded by *aadA1* and *aadA*2, acetyltransferases by *aac(6″)-Ib* and *aacA4* genes, and phosphorylases by *aph(6)-Id*, *aph(6)-Ib*, and *aph(6)-Ia* genes [3]. Panaminoglycoside resistance that was associated with 16S rRNA methylase was also reported in *P. mirabilis* [3]. *P. mirabilis* develops resistance to fluoroquinolones by mutating the genes encoding DNA gyrase (*gyr*A) and topoisomerase (*par*C) or acquiring plasmid-mediated *qnr* genes encoding qnr proteins, which protect topoisomerase IV. Furthermore, *sul1* and *sul2* genes usually confer resistance to sulfonamides, while *dfr* genes encode dihydropholate reductase, antagonizing the effect of trimethoprim [3].

Bibliographical data on the β-lactam resistance mechanisms in *P. mirabilis* primarily focus on ESBLs and p-AmpC. In the early 1990s, ESBLs in *P. mirabilis* were predominantly associated with the TEM enzyme family; however, a shift toward the CTX-M family was observed in the early 2000s [3]. CMY is almost the only p-AmpC family reported in *P. mirabilis* [3]. In Croatia, an outbreak of infections with TEM-52 β-lactamase-producing *P. mirabilis* was reported in Split in 2008 [8]. Subsequently, an outbreak of infections with CMY-16 was described in a nursing home in Zagreb [9]. The same allelic variant was much later identified in a hospital in Split [10]. The widespread use of carbapenems in the treatment of infections with ESBL and p-AmpC-producing organisms leads to the proliferation of carbapenemases in *P. mirabilis*. The majority of studies detected carbapenemases that belonged to the VIM and NDM families and as well as OXA-48 [3]. However, there are no reports that address carbapenemases in *P. mirabilis* isolates from Croatia so far.

In this study, we report on an outbreak of extensively drug resistant (XDR) carbapenemase-producing *P. mirabilis* in a psychiatric hospital in Zagreb. We aimed to characterize β-lactamases and other resistance mechanisms that are carried by these isolates and their molecular epidemiology.

## 2. Materials and Methods

### 2.1. Patients and Bacterial Isolates

The bacterial isolates with reduced susceptibility to at least one carbapenem (imipenem, meropenem, or ertapenem) were collected from 2023 to 2024 from a psychiatric hospital in Zagreb. The taxonomy was investigated using MALDI-TOF MS (matrix-assisted laser desorption ionization–time of flight mass spectrometry) (Vitek MS Biomerieux, Marcy-l’Étoile, France). The initial antibiotic susceptibility was tested according to the EUCAST guidelines [11] in the Dr. Andrija Štampar Teaching Institute of Public Health, which provides microbiological service for the psychiatric hospital, “Sveti Ivan”. Isolates that demonstrated reduced susceptibility to at least one carbapenem were sent to the Clinical Department for Clinical Microbiology and Infection Control and Prevention of the University Hospital Centre Zagreb for further analysis.

### 2.2. Antimicrobial Susceptibility Testing

The broth dilution method was applied to determine the minimum inhibitory concentrations (MICs) of 13 antibiotics: amoxicillin alone and with clavulanic acid, piperacillin–tazobactam, cefuroxime, ceftazidime, cefotaxime, ceftriaxone, cefepime, imipenem, meropenem, gentamicin, amikacin, and ciprofloxacin. The results were interpreted following the CLSI guidelines [12]. The susceptibility to ceftazidime–avibactam, sulphametoxazole–trimethoprim, and chloramphenicol was determined only by disk-diffusion test. The antibiotic-containing disks were provided by Oxoid (Basingstoke, UK). The antibiotics that demonstrate intrinsic resistance in *Proteus* spp., such as colistin, tetracycline, and tigecycline, were not tested. The isolates were classified as multidrug resistant (MDR), extensively drug resistant (XDR), or pandrug resistant (PDR), as described previously by Magiorakos et al. [13]. MDR is recognized as being non-susceptible to at least one antimicrobial agent in three antimicrobial classes. XDR is recognized as being resistant to at least one antimicrobial agent in all antimicrobial classes and susceptible to only two or fewer. The PDR isolates were resistant to all antibiotics available for the specific species. Antibiotics with intrinsic, chromosomal resistance were excluded (nitrofurantoin, tigecycline, tetracycline, colistin). *Escherichia coli* ATCC 25922 and *Klebsiella pneumoniae* 700603 served as quality control strains for MIC determination.

### 2.3. Phenotypic Detection of β-Lactamases

ESBLs were detected by a double-disk synergy test (DDST) [14] and combined disk test with cephalosporins and clavulanic acid [12]. Plasmid-mediated AmpC β-lactamases were detected among cefoxitin-resistant isolates by a combined disk test using cephalosporin disks combined with cloxacillin as an inhibitor [15]. An initial screening for carbapenemases was carried out for the purpose of routine microbiology testing by an immunochromatographic test [16]. A modified Hodge test (MHT) with an imipenem disk was used to confirm the production of carbapenemases [17]. Since the identification of carbapenemases in *P.mirabilis* poses an identification challenge due to low levels of resistance in this species, additional tests were conducted. The carbapenem inactivation method (CIM) was applied to assess for the hydrolysis of carbapenems with disks of imipenem and meropenem [18]. A suspension of the test strain was adjusted to McFarland 0.5 (10^8^ CFU/mL), and a meropenem disk was placed in the suspension. The suspension was incubated for 2 h at 37 °C. *E. coli* ATCC 25922 was inoculated on Mueller–Hinton agar (MH), and the disk was placed in the middle of the plate. The plates were incubated overnight, and the lack of inhibition zone, decreased inhibition zone (<15 mm), or colonies within the inhibition zone indicated carbapenem hydrolysis. Isolates that tested positive in the CIM test were further analyzed by EDTA-CIM (eCIM) to determine the presence of an MBL. The eCIM test was performed in the same way, but with one tube containing 0.5 mM EDTA to inhibit the MBLs. The test indicated MBL positivity if there was a ≥5 mm increase in zone diameter in the eCIM experiment compared to the control sample without EDTA [18]. Due to the high rate of CIM negativity among the isolates that tested positive using the immunochromatographic assay, additional confirmatory testing was performed by using the combined disk-diffusion methods. Disks containing imipenem and meropenem alone as well as combined with PBA, 0.1 M EDTA, or both were used to detect KPC, MBLs, or the simultaneous production of KPC and MBL, respectively [17]. The strains from our own collection, known to be positive for KPC, VIM, NDM, and OXA-48, were used as positive and negative controls.

### 2.4. Molecular Detection of Resistance Genes

DNA was extracted by thermal lysis at 100 °C for 10 min. Cellular debris was removed by centrifugation, and the supernatant was used as the DNA template. Antimicrobial resistance (AMR) genes comprising β-lactam (*bla*_TEM_, *bla*_SHV_, and *bla*_CTX-M_) [19,20,21] and fluoroquinolones (*qnr*A, *qnr*B, and *qnr*S) [22] were assessed by the singleplex PCR, as described previously. Carbapenemase-coding genes belonging to class A (*bla*_KPC_), B, MBLs (*bla*_IMP_, *bla*_VIM_, and *bla*_NDM_), class D (*bla*_OXA-48_, [23], *bla*_OXA-23_, *bla*_OXA-24/40_, *bla*_OXA-51_, and *bla*_OXA-58_) [24], *bla*_AmpC_ [25], and the genes of five clonal groups of CTX-M β-lactamases were amplified via multiplex PCR according to the protocols reported previously [26]. The selected amplicons (11 CTX-Ms and 8 VIMs) obtained by the singleplex PCR were sequenced with forward and reverse primers by employing the Sanger method using the Eurofin Genomic service (https://eurofinsgenomics.eu, assessed on 5 February 2025), and the sequences were compared with those from the GenBank nucleotide database at http://www.ncbi.nlm.nih.gov/blast (accessed on 1 February 2025). The flanking regions of the *bla*_CTX-M_ genes were investigated by PCR mapping by using the forward primer for IS*Ecp* or IS*26*, combined with the universal reverse primer for the CTX-M-encoding genes (MA3) [27].

### 2.5. Inter-Array Genotyping Kit CarbaResist

The genotyping of six randomly selected isolates was conducted using the microarray-based CarbaResist Genotyping Kit, according to the manufacturer’s instructions, version 1012012100004 (INTER-ARRAY, fzmb GmbH, Bad Langensalza, Germany). In short, genomic DNA was isolated from monoclonal overnight cultures using the Qiagen DNeasy Blood and Tissue Kit, according to the manual. The unfragmented DNA was linear amplified using one primer for each target sequence (antisense) and was internally labeled with biotin dUTP. The obtained ssDNA (single-stranded) products were transferred into the ArrayWells for hybridization. These wells contained 230 probes, corresponding to distinct genes for the most relevant carbapenemases, ESBL and AmpC, as well as genes associated with β-lactam, aminoglycoside, fluoroquinolone, sulphonamide, trimethoprim, and colistin resistance. After the washing steps were completed to remove any unbound DNA, horseradish peroxidase (HRP)-conjugated streptavidin was bound to all of the hybridized sections, resulting in dark spots on the chip due to an enzymatic reaction. The detection of these spots and data acquisition was performed automatically using the INTER-VISION Reader.

### 2.6. Whole-Genome Sequencing (WGS)

Four randomly selected representative isolates were subjected to the WGS. First, the strains were cultivated in Tryptic Soy Broth (Merck Millipore, MA, Burlington USA) at 37 °C overnight. Then, the genomic DNA was extracted using the QIAamp DNA Mini Kit (Qiagen, Hilden, Germany) according to the manufacturer’s instructions. Subsequently, the extracts were barcoded via the Rapid Barcoding Kit 96 V14 and sequenced using the minION (Oxford Nanopore Technologies). For the assembly of the single reads, Flye (https://www.pnas.org/doi/full/10.1073/pnas.1604560113, accessed on 20 June 2025) was utilized. Subsequently, the data were analyzed using the webservers and services of the Center for Genomic Epidemiology (http://www.genomicepidemiology.org, accessed on 20 June 2025) [28]. The phylogenetic tree was generated with the REALPHY online tool (https://doi.org/10.1093/molbev/msu088, accessed on 20 June 2025) and visualized using phylo.io (https://doi.org/10.1093/molbev/msw080, accessed on 20 June 2025). The phylogenetic tree was constructed using WGS for the purpose of genotyping in order to determine the genetic relatedness between isolates. Online servers were used. To build the tree, a maximum likelihood method (PhyML) was used, where the SNPs and nonpolymorphic sites were incorporated for improved accuracy. To assess relatedness, the default parameters were used to balance efficiency and accuracy. The detailed criteria for relatedness can be found under the link provided at the link above. Plasmid replicons were investigated using Plasmd Finder.

### 2.7. Characterization of Plasmids

Conjugation experiments were carried out by the broth-based method [29]. Donor and recipient strains were grown to the exponential stage, mixed in a ratio of 1:1, and incubated overnight at 37 °C. Transconjugants were selected on MacConkey agar containing imipenem or cefotaxime to inhibit the recipient strain and sodium azide so as to suppress the donor isolates, were selected. PCR-based replicon typing (PBRT) was performed according to Carattoli et al. [30]. Since it was observed previously that PBRT can be inefficient in identifying the L/M plasmid incompatibility type, an updated method designated to identify and distinguish between IncL and IncM plasmids was applied [31]. Positive control strains were kindly provided by Dr. Alessandra Carattoli, Instituto Superiore di Sanita, Rome, Italy.

### 2.8. Detection of Virulence Determinants

Urease activity was determined by inoculating the strains in the urea-containing medium. The change in the color to pink was recorded as a positive result. The haemolytic activity was tested by culturing the strains on a 10% sheep blood plate with the addition of trimethoprim to inhibit swarming. For the motility assay, one colony was stabbed with a one-microliter loop into a semisolid nutrient medium. After overnight incubation at 37 °C, motility was measured as turbidity of the medium [2].

## 3. Results

### 3.1. Patients and Bacterial Isolates

In total, 20 isolates with reduced susceptibility to at least one carbapenem were isolated from 3 November 2023 until 24 July 2024. All isolates originated from the urinary tract and all except two from urinary catheters (Supplementary Material Pathogens 3754486 Table S1). All patients were females with ages ranging from 49–90 years (the median age was 79).

### 3.2. Antibiotic Susceptibility

The antimicrobial susceptibility results for the 20 *P. mirabilis* isolates are listed in Table 1. They all exhibited identical resistance profiles with MICs of amoxicillin alone., combined with clavulanic acid, cefuroxime, cefotaxime, ceftriaxone, cefepime, gentamicin, amikacin, and ciprofloxacin, exceeding 128 mg/L in the majority of isolates, indicating high-level resistance. Regarding carbapenems, the isolates displayed reduced susceptibility towards imipenem, with variable MIC values ranging from 4 to 128 mg/L, but they demonstrated susceptibility to meropenem and ertapenem. A high resistance rate of 65% (13/20) was observed for ceftazidime. (Table 1) Piperacillin–tazobactam retained activity, with all isolates being susceptible according to the CLSI with a MIC ≤ 16 mg/L. However, if the EUCAST criteria were to be applied with a lower resistance breakpoint (16 mg/L), then 40% (*n* = 8) of the isolates would be resistant. On the other hand, according to the EUCAST criteria, only four isolates were resistant to imipenem (40%). All but one isolate showed susceptibility to cefiderocol. All isolates were classified as XDR. Meropenem, ertapenem, and cefiderocol exerted high activity, with MICs of meropenem below 0.5 mg/L.

### 3.3. Phenotypic Detection of β-lactamases

Based on the DDST and combined disk tests using clavulanic acid, all isolates were ESBL positive. All isolates were susceptible to cefoxitin, indicating the lack of p-AmpC. Regarding carbapenemases, the Hodge test was negative in all except for one isolate (95%), as no distortion of the inhibition zone around the imipenem disk was seen. Furthermore, CIM and eCIM were positive in 85% of the isolates (*n* = 17), indicating the production of an MBL, but only with the imipenem disk (Supplementary Table S1). The rate of positivity with the meropenem disk was only 35% (*n* = 7). However, the inhibitor-based disk test with the EDTA yielded positive results in 65% (*n* = 13), again only with the imipenem disk, thus raising suspicion of an MBL (Supplementary Table S1).

### 3.4. Molecular Detection of Resistance Genes

The PCR revealed *bla*_CTX-M_ genes belonging to cluster 1, *bla*_TEM_ and *bla*_VIM_ genes in all tested isolates. The sequencing of eight randomly selected VIM amplicons identified one *bla*_VIM-1,_ *bla*_VIM-4,_ and *bla*_VIM-78_ genes respectively, and five *bla*_VIM-75_ genes. All 11 CTX-M amplicons encoded the CTX-M-15 allelic variant. Furthermore, *qnr* genes for fluoroquinolone resistance were not detected, and the IS*Ecp* insertion element was linked to the *bla*_CTX-M_ genes.

### 3.5. Genotyping by Inter-Array Genotyping Kit CarbaResist

The genomes of all the tested isolates possessed almost identical resistance gene contents, including the *bla*_TEM_ and *bla*_CTX-M-15_ genes associated with the IS*Ecp* insertion element, *bla*_VIM_ providing β-lactam resistance, *aac(*6)-*IIc*, *aadA1,* and *aadA2* coding enzymes for aminoglycoside modification, *armA* for ribosomal methylase, *sul1* and *sul2* for sulphonamide resistance, and *dfrA1* for dihydrofolate reductase, rendering trimethoprim resistant, as shown in Table 2. One isolate harbored the *dfrA15* allelic variant (Table 2). All isolates harbored integrase genes for class 1 and/or class 2 integrons.

### 3.6. WGS and Plasmid Analysis

All four tested isolates presented with almost identical AMR genes: *bla*_CTX-M-202_, *bla*_TEM-2_, *bla*_TEM-1A_, and *bla*_VIM-4_ for β-lactam resistance; *aac(3)-IId*, *aph(6)-Id*, *aph(3″)-Ib*, *aadA1*, *armA*, and *aac(6′)-IIc* for aminoglycoside resistance; *sul1* and *sul2* for sulphonamide resistance; *dfrA1* encoding dihydropholate reductase; *cat* for chloramphenicol acetyltransferase; and *tet(J)* responsible for tetracycline resistance, as shown in Table 3. Two isolates possessed duplicate VIM genes: *bla*_VIM-1_ and *bla*_VIM-4_. Fluoroquinolone resistance genes were not detected. The multiplex PCR, according to Carattoli et al., did not detect any typable plasmid, but the WGS identified IncQ1 plasmid in selected representative isolates. An analysis of the integrons further identified gene cassettes with β-lactam, aminoglycoside, sulphonamide, and trimethoprim resistance genes in all of them, except for one tested isolate. Only in isolate 6 (41149/-24) it was seen that two resistance genes could not be annotated to the integron regions. No transconjugants were obtained after repeated attempts.

### 3.7. Detection of Virulence Determinants

All isolates were positive for urease activity, hemolysis, and motility.

### 3.8. Genotyping (Phylogenetic Tree)

**Figure 1:**
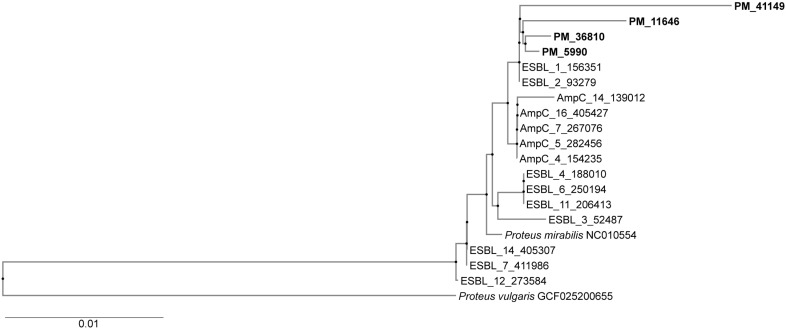
Phylogenetic tree showing genetic relatedness between isolates. Four isolates from the present study showed a high level of genetic relatedness but no similarity with ESBL and p-AmpC-producing organisms from the previous study. PM3-36810, PM5-5990, PM6-41149, and PM8-11646.

Genotyping demonstrated that four representative isolates were clonally related but distinct from ESBL and p-AmpC-positive isolates from a previous study, as shown in Figure 1. According to the WGS, three representative isolates, PM 3, PM 5, and PM 8, were found to belong to ST 135.

## 4. Discussion

This study described an outbreak of UTIs with VIM-1-positive *P. mirabilis*. We characterized the resistance determinants and molecular epidemiology of the isolates. All isolates had identical resistance patterns and genes. This study showed development of resistance of this important hospital pathogen and its ability to accumulate β-lactam resistance determinants. The bibliographical references on MBLs in *P. mirabilis* are scarce and mostly reported from the Far East [32]. Furthermore, Europe, Greece, and Bulgaria are focal points of VIM-producing *P. mirabilis* [33,34,35,36]. A sporadic occurrence was recorded in Germany [37]. Our isolates were resistant to imipenem but susceptible to ertapenem and meropenem and, thus, represent a hidden reservoir of VIM MBLs in *Enterobacterales*. The use of molecular methods and immunochromatographic tests could help to uncover the underestimated carriage of VIM-producing *P. mirabilis* since the isolates remained susceptible to two out of three carbapenems available in Croatia. Currently, there are about 80 allelic variants of VIM MBLs, but the most reported types are VIM-1 and VIM-4. This is the first time that the VIM-75 and VIM-78 allelic variants were reported in Croatia. They were previously detected among XDR *P. mirabilis* isolates from Germany [37]. Similarly, as in Germany, two of our tested isolates harbored duplicate VIM carbapenemases (VIM-1 and VIM-4). In contrast to the wide variety of VIM allelic variants, all CTX-M amplicons encoded CTX-M-15, the most widespread allelic variant of the CTX-M enzymes. All isolates were categorized as XDR, as they were susceptible only to meropenem, ertapenem, and cefiderocol. However, the resistance phenotype is dependent on what criteria were applied. According to the CLSI, all isolates were susceptible to piperacillin–tazobactam and resistant to imipenem; however, if the MICs were interpreted using the EUCAST recommendations, which are used in routine diagnostics in Croatia, all isolates would be resistant to piperacillin–tazobactam and the majority intermediately susceptible to imipenem (susceptible at increased exposure). The isolates exhibited variable levels of resistance to expanded-spectrum cephalosporins and carbapenems with imipenem MICs ranging from 4 to >128 mg/L. This could be due to the variable expression of the *bla*_VIM_ genes. Here, we have, for the first time, characterized duplicate carbapenemases in *P. mirabilis.* In our previous studies, they were reported in *K. pneumoniae* [38], but the most common combination was NDM+OXA-48. This is the first time that the combination of two MBLs has been reported in Croatia. IS*26* was found to upregulate the *bla*_VIM_ gene and increase the level of resistance [33,34]; however, this was not found in our study, which could explain the very low MICs of carbapenems.

The MBL encoding genes were embedded into the class 1 or class 2 integrons, which could be transferred and integrated into the plasmids or chromosomes. These integrons are responsible for the dissemination of the gene cassettes carrying resistance determinants to β-lactam antibiotics, aminoglycosides, and sulphonamides, similar to those found in Germany [37]. In contrast to our results, CHDL, previously identified only in *Acinetobacter baumannii*, was dominant in *P. mirabilis* in France, Belgium, and Germany. Similarly, as MBLs, they pose a diagnostic challenge due to their high susceptibility to carbapenems [39,40,41]. In contrast to other *Enterobacterales*, VIM positivity affected only imipenem susceptibility and rendered it resistant. Meropenem and ertapenem remained susceptible.

CTX-M-15 ESBL was detected as additional β-lactamases to VIM. It conferred high-level resistance to third- and fourth-generation cephalosporins as well as to amoxicillin–clavulanate on the producing isolates. IS*Ecp* upstream of *bla*_CTX-M_ gene acted as a promoter and increased the expression of the gene. The CTX-M-202 allelic variant, detected by WGS, is very rare. It belongs to the CTX-M-1 cluster and is related to CTX-M-15 [42].

In spite of very high MICs of fluoroquinolones, there were no plasmid-born resistance genes and, thus, resistance is most likely due to the mutations of the *gyr*A and *par*C genes. High-level resistance to aminoglycosides is in line with abundant AMR genes encoding acetylases, adenylases, and phosphorylases, which modify aminoglycosides and render them inactive. The presence of *sul*, *tet*, and *cat* genes is in concordance with sulphonamide, tetracycline, and chloramphenicol resistance, respectively. Interestingly, there were only two allelic variants of the *dfr* gene coding for the dihydropteroate synthase.

Unfortunately, the phenotypic tests based on carbapenem hydrolysis proved unreliable. The MHT detected only one isolate as a carbapenemase producer, and the CIM exhibited positive results only with the imipenem disk. This is due to a low expression of *bla*_VIM_ genes and weak carbapenem hydrolysis. The phenotypic detection of MBLs is mostly based on the carbapenem-EDTA synergy test. In the present study, we performed two forms of tests: the combined disk test with EDTA and eCIM, but both exerted moderate sensitivity, visible only with the imipenem disk. A similar problem was observed with OXA-23 and OXA-58 producing isolates in France and Belgium [39,40,41]. Apparently, expression of the carbapenemase-encoding genes is very weak among the *Proteus* spp. isolates regardless of the carbapenemase type. On the contrary, the immunochromatographic test showed high concordance with the PCR, WGS, and Inter-Array Kit CarbaResist. All four methods detected the VIM-resistant determinant in all tested isolates. Some researchers recommended the use of dipicolinate as a metal chelator for *Proteus* spp., which provides better sensitivity compared to EDTA [33]. However, the compound is not available in most routine laboratories.

Among *Enterobacterales*, *K. pneumoniae* and *E. coli* are typical hospital pathogens that bear the *bla*_CARB_ genes that are associated with hospital outbreaks. However, *P. mirabilis* is gaining importance as a nosocomial pathogen and with outbreaks due to ESBL, p-AmpC, and carbapenemase-producing isolates that are being reported worldwide. In Croatia, two hospital outbreaks were reported: one with TEM-52-producing *P. mirabilis*, and the other with CMY-16-producing *P. mirabilis*, both from Split [8,10]. The therapeutic options are very limited, which is also due to an intrinsic resistance to tigecycline and colistin. Fortunately, cefiderocol susceptibility tested positive in all except for one strain. There was no resistance recorded to meropenem and ertapenem. However, in case of carbapenem administration, there is a risk of developing mutants that hyperproduce VIM β-lactamase under selection pressure.

There are certain limitations to our study. Unfortunately, we cannot explain if there was a vertical or horizontal transmission of the resistance genes because conjugation was unsuccessful, and WGS was only conducted on a small number of isolates. The IncC plasmid type was found in all tested isolates, but there is no evidence of it harboring resistance genes. In the previous studies, IncA/C or IncL/M were found to carry the *bla*_VIM_ genes.

The similarity between isolates detected by WGS indicated common ancestry. The fact that all isolates possessed identical AMR genes points towards a common source. The phylogenetic tree also confirmed high genetic relatedness between the isolates, but there was no similarity to the older ESBL and p-AmpC positive isolates, which could indicate that the MBL-positive organisms did not evolve from the previous isolates, but developed de novo. The described MDR clone successfully spread within the two women’s wards of a psychiatric hospital, causing urinary infections in catheterized patients. The hospital outbreak was terminated after the introduction of measures to prevent the spread of the VIM-positive *P. mirabilis* clone. However, subsequent UTIs caused by this clone were detected in two nursing homes in Zagreb because the patients were transferred from the hospital. Thanks to the implemented prevention measures, the clone did not spread within those two nursing homes. The screening for MDR bacteria should be done if there is a transfer from a hospital to a long-term care facility; however, there are many private nursing homes in Zagreb, and surveillance cultures are not taken in all of them.

## 5. Conclusions

An analysis of local antimicrobial resistance patterns is necessary in order to avoid hospital outbreaks with difficult-to-treat isolates. Hospital hygiene measures should be implemented to limit the spread of MDR *P. mirabilis*. This is the first report of carbapenemase among *P. mirabilis* in Croatia. All isolates originated from the urinary tract, emphasizing the significance of UTIs as a reservoir of resistant bacteria. The isolates exhibited identical resistance phenotype and gene contents, pointing to a common source. The implementation of new molecular methods enabled the detection of a plethora of resistance genes for various antibiotic classes. Compared to the Inter-Array Genotyping Kit CarbaResist method, WGS identified more resistance genes and provided allelic variants of the genes, which is important for analysis in molecular epidemiology.

## Figures and Tables

**Table 1:** Antibiotic susceptibility profiles of VIM-bearing *P. mirabilis* isolates to different antibiotics.

	AMX 32	AMC 32/16	TZP 32/4	CXM 32	CAZ 16	CTX 4	CRO 4	FEP 16	IMI 4	MEM 4	GM 16	CIP 0.25
1	>128 (R)	32/16(R)	8/4(S)	>128(R)	8(I)	>128(R)	16(R)	16(R)	128(R)	0.25(S)	>128(R)	>128(R)
2	>128 (R)	64/32(R)	16/4(S)	>128(R)	8(I)	>128(R)	16(R)	>128(R)	128(R)	0.25(S)	>128(R)	>128(R)
3	>128 (R)	64/32(R)	8/4(S)	>128(R)	16(R)	>128(R)	32(R)	32(R)	128(R)	0.25(S)	>128(R)	>128(R)
4	>128 (R)	128/64(R)	8/4(S)	>128(R)	2(S)	>128(R)	>128(R)	>128(R)	128(R)	0.25(S)	>128(R)	>128(R)
5	>128 (R)	32/16(R)	16/4(S)	>128(R)	2(S)	>128(R)	>128(R)	>128(R)	4(R)	0.25(S)	>128(R)	>128(R)
6	>128 (R)	32/16(R)	16/4(S)	>128(R)	8(I)	>128(R)	>128(R)	>128(R)	4(R)	0.25(S)	>128(R)	>128(R)
7	>128 (R)	128/64(R)	8/4(S)	>128(R)	8(I)	>128(R)	>128(R)	>128(R)	4(R)	0.25(S)	>128(R)	>128(R)
8	>128 (R)	128/64(R)	8/4(S)	>128(R)	16(R)	>128(R)	>128(R)	>128(R)	8(R)	0.25(S)	>128(R)	>128(R)
9	>128 (R)	128/64(R)	8/4(S)	>128(R)	16(R)	>128(R)	64(R)	>128(R)	4(R)	0.25(S)	>128(R)	>128(R)
10	>128 (R)	128/64(R)	8/4(S)	>128(R)	32(R)	>128(R)	>128(R)	>128(R)	4(R)	0.25(S)	>128(R)	>128(R)
11	>128 (R)	128/64(R)	8/4(S)	>128(R)	128(R)	>128(R)	32(R)	>128(R)	4(R)	0.25(S)	>128(R)	>128(R)
12	>128 (R)	32/16(R)	16/4(S)	>128(R)	2(S)	>128(R)	>128(R)	>128(R)	8(R)	0.25(S)	>128(R)	>128(R)
13	>128 (R)	128/64(R)	8/4(S)	>128(R)	16(R)	>128(R)	32(R)	>128(R)	8(R)	0.25(S)	>128(R)	>128(R)
14	>128 (R)	128/64(R)	16/4(S)	>128(R)	32(R)	>128(R)	64((R)	>128(R)	4(R)	0.25(S)	>128(R)	>128(R)
15	>128 (R)	128/64(R)	8/4 (S)	>128(R)	32(R)	>128(R)	16(R)	>128(R)	4(R)	0.25(S)	>128(R)	>128(R)
16	>128 (R)	128/64(R)	16/4(S)	>128(R)	128(R)	>128(R)	128(R)	>128(R)	4(R)	0.12(S)	>128(R)	>128(R)
17	>128 (R)	64/32(R)	16/4(S)	>128(R)	16(R)	>128(R)	32(R)	>128(R)	8(R)	0.06 (S)	>128(R)	>128(R)
18	>128 (R)	63/32(R)	8/4(S)	>128(R)	64(R)	>128(R)	64(R)	>128(R)	4(R)	0.25(S)	>128(R)	>128(R)
19	>128 (R)	128/64(R)	8/4(S)	>128(R)	64(R)	>128(R)	32(R)	>128(R)	4(R)	0.25(S)	>128(R)	>128(R)
20	>128 (R)	64/32(R)	16/4(S)	>128(R)	64(R)	>128(R)	128(R)	>128(R)	4(R)	0.5(S)	>128(R)	>128(R)

Abbreviations: AMX—amoxicillin; AMC—amoxicillin/clavulanic acid; TZP—piperacillin–tazobactam; CXM—cefuroxime; CAZ—ceftazidime; CTX—cefotaxime; CRO—ceftriaxone; FEP—cefepime; IMI—imipenem; MEM—meropenem; GM—gentamicin; CIP—ciprofloxacin; R—resistant; I—Intermediate susceptible (susceptible at increased exposure); S—susceptible. Resistance breakpoint is provided (mg/L).

**Table 2:** Resistome of *P. mirabilis* isolates by Inter-Array Kit CarbaResist.

Isolate and Protocol Number	Res Phenotype	β-Lactam	Aminoglycoside	Sulphamide	Trimethoprim	Integrase Genes
PM 2	AMX, AMC, TZP, CAZ, CTX, CRO, FEP, GM, AMI CIP	*ISEcpbla*_CTX-M-15_ *bla*_TEM_ *bla*_VIM_	*aac(6″)IIc* *aadA1* *aadA2* *armA*	*sul1*	*dfrA1*	*Intl2*
PM 3	AMX, AMC, TZP, CAZ, CTX, CRO, FEP, GM, AMI CIP	*ISEcpbla*_CTX-M-15_ *bla*_TEM_ *bla*_VIM_	*aac(6″)IIc* *aadA1* *aadA2* *armA*	*sul1* *sul2*	*dfrA1*	*Intl1* *Intl2*
PM4	AMX, AMC, TZP, CAZ, CTX, CRO, FEP, GM, AMI CIP	*ISEcpbla*_CTX-M-15_ *bla*_TEM_ *bla*_VIM_	*aac(6″)IIc* *aadA1* *aadA2* *armA*	*sul1* *sul2*	*dfrA1* *dfrA15*	*Intl1* *Intl2*
PM11	AMX, AMC, TZP, CAZ, CTX, CRO, FEP, GM, AMI CIP	*ISEcpbla*_CTX-M-15_ *bla*_TEM_ *bla*_VIM_	*aac(6″)IIc* *aadA1* *aadA2* *armA*	*sul1* *sul2*	*dfrA1*	*Intl1* *Intl2*
PM14	AMX, AMC, TZP, CAZ, CTX, CRO, FEP, GM, AMI CIP	*ISEcpbla*_CTX-M-15_ *bla*_TEM_ *bla*_VIM_	*aac(6″)IIc* *aadA1* *aadA2* *armA*	*sul1* *sul2*	*dfrA1*	*Intl1* *Intl2*
PM 19	AMX, AMC, TZP, CAZ, CTX, CRO, FEP, GM, AMI CIP	*ISEcpbla*_CTX-M-15_ *bla*_TEM_ *bla*_VIM_	*aac(6″)IIc* *aadA1* *aadA2* *armA*	*sul1* *sul2*	*dfrA1*	*Intl1* *Intl2*
PM 20	AMX, AMC, TZP, CAZ, CTX, CRO, FEP, GM, AMI, CIP	*ISEcpbla*_CTX-M-15_ *bla*_TEM_ *bla*_VIM_	*aac(6″*)*IIc* *aadA1* *aadA2* *armA*	*sul1* *sul2*	*dfrA1*	*Intl1* *Intl2*

**Table 3:** Whole-genome sequencing of representative isolates. Accession numbers are provided in a separate section at the end of the manuscript.

Isolate and Protocol Number	β-Lactam	Aminoglycosides	Sulphonamide	Trimethoprim	Chloramphenicol	Tetracycline	Plasmid Replicon
PM 3	*bla*_CTX-M-202_, *bla*_TEM-156_, *bla*_TEM-1A_, *bla*_TEM-2_, *bla*_VIM-4_, *bla*_VIM-1_,	*aac(3)-IId*, *aph(6)-Id*, *aph(3″)-Ib*, *aadA1*, *armA*, *aac(6′)-IIc*,	*sul1* *sul2*	*dfrA1*	*cat*	*tet(J)*	*IncC*
PM 5	*bla*_CTX-M-202_, *bla*_TEM-2_, *bla*_TEM-1A_, *bla*_VIM-4_	*aac(3)-IId*, *aph(6)-Id*, *aph(3″)-Ib*, *aadA1*, *armA*, *aac(6′)-IIc*,	*sul1* *sul2*	*dfrA1*	*cat*	*tet(J)*	*IncC*
PM 6	*bla*_CTX-M-202_, *bla*_TEM-2_, *bla*_VIM-4_	*aph(6)-Id*, *aph(3″)-Ib*, *armA*, *aac(6′)-IIc*, *aac(3)-IId*, *aac(6′)-IIc*, *aadA1*	*sul1* *sul2*	*dfrA1*	*cat*	*tet(J)*	*IncC*
PM 8	*bla*_CTX-M-202_, *bla*_TEM-2_, *bla*_TEM-1A_, *bla*_VIM-1_, *bla*_VIM-4_	*aac(3)-IId*, *aph(6)-Id*, *aph(3″)-Ib*, *aadA1*, *armA*, *aac(6′)-IIc*,	*sul1* *sul2*	*dfrA1*	*cat*	*tet(J)*	*IncC*

## Data Availability

The data presented in this study are available on request from the corresponding author. All information about the strains is given in Supplementary Tables S1 and S2. Resistance gene sequences were deposited in the NCBI Gene bank with the following accession numbers: *aac(3)-Iid*, EU022314; *aph(6)-Id*, M28829; *aph(3″)-Ib*, AF321551; *aadA1*, JQ480156; *armA*, AY220558; *aac(6′)-Iic*, NC_012555; *bla*_CTX-M-202_, MF195067; *bla*_TEM-1A_, HM749966; *bla*_VIM-4_, EU581706; *bla*_VIM-1_, Y18050; *bla*_TEM-2_, X54606; *cat*, M11587; *sul1*, U12338; *sul2*, FN995456; *tet(J)*, ACLE01000065; *dfrA1*, X00926.

## Supplementary Materials

The following supporting information can be downloaded at: https://www.mdpi.com/article/10.3390/pathogens14080737/s1, Table S1: clinical and epidemiological data, antibiotic susceptibility, PCR results, and plasmid replicons of the isolates.
