# Supplementary material for: Outbreak Caused by VIM-1- and VIM-4-Positive Proteus mirabilis in a Hospital in Zagreb
Source: Pathogens. 2025 Jul 26;14(8):737. doi: 10.3390/pathogens14080737 (PMC12389321; doi:10.3390/pathogens14080737)
Supplement: Supplementary file 1 [file pathogens-14-00737-s001.zip › pathogens-3754486-supplementary.pdf]

Table S1

		age	gender	date	specimen	AMX	AMC	CN	CXM	CAZ	CTX	CRO	FEP	IMI	MEM	ERT	GM
1	75791/2/23	84	F	29.12. 2023.	URINE (CAT)	R	R	R	R	R	R	R	R	R	S	S	R
2	62844/2/23	58	F	03. 11. 2023.	URINE (CAT)	R	R	R	R	R	R	R	R	R	S	S	R
3	36810/2/24	74	F	12.06. 2024.	URINE (CAT)	R	R	R	R	R	R	R	R	R	S	S	R
4	75296/2/23	73	F	05.01.2024.	URINE (CAT)	R	R	R	R	R	R	R	R	R	S	S	R
5	5990/2/24	73	F	29.01.2024.	URINE (CAT)	R	R	R	R	R	R	R	R	R	S	S	R
6	41149/2-24	84	F	03.07. 2024.	URINE (CAT)	R	R	R	R	R	R	R	R	R	S	S	R
7	6714	89	F	12.06. 2024.	URINE (CAT)	R	R	R	R	R	R	R	R	R	S	S	R
8	11646/1-24	68	F	20. 02. 2024.	URINE (CAT)	R	R	R	R	R	R	R	R	R	S	S	R
9	189/2-24	81	F	06.01. 2024.	URINE	R	R	R	R	R	R	R	R	R	S	S	R
10	43529/2-24	67	F	16.07.2024,	URINE (CAT)	R	R	R	R	R	R	R	R	R	S	S	R
11	40567/2/24	76	F	01.07. 2024.	URINE (CAT)	R	R	R	R	R	R	R	R	R	S	S	R
12	871-2-24	84	F	3.7.2024	URINE (CAT)	R	R	R	R	R	R	R	R	R	S	S	R
13	70017/2023-2	85	F	30. 11. 2023.	URINE	R	R	R	R	R	R	R	R	R	S	S	R
14	44350/2-24	89	F	22.07. 2024.	URINE (CAT)	R	R	R	R	R	R	R	R	R	S	S	R
15	62846/2-23	86	F	03. 11. 2023.	URINE (CAT)	R	R	R	R	R	R	R	R	R	S	S	R
16	15588/2/24	49	F	07.03. 2024.	URINE (CAT)	R	R	R	R	R	R	R	R	R	S	S	R
17	13600/2-24	68	F	26.02. 2024.	URINE (CAT)	R	R	R	R	R	R	R	R	R	S	S	R

18	44800/2/24	90	F	24.07. 2024.	URINE (CAT)	R	R	R	R	R	R	R	R	R	S	S	R
19	9124/2-24	80	F	09.02. 2024.	URINE	R	R	R	R	R	R	R	R	R	S	S	R
20	133347	79	F	01.03. 2024.	URINE (CAT)	R	R	R	R	R	R	R	R	R	S	S	R
